# First report of *Plum bark necrosis stem pitting-associated virus* infecting grapevine in China

**DOI:** 10.1186/s12985-020-01438-3

**Published:** 2020-11-18

**Authors:** Yang Yang, Yifan Sun, Qingliang Li, Yufeng Wu, Deya Wang

**Affiliations:** 1 College of Agriculture, Nanjing Agriculture University, Nanjing, 210095 People’s Republic of China; 2 College of Life Sciences, Zaozhuang University, Zaozhuang, 277160 People’s Republic of China

**Keywords:** Grapevine, *Grapevine virus A*, *Plum bark necrosis stem pitting-associated virus*, China

## Abstract

**Background:**

Virus disease is one of the main diseases in grapevine, and there has been no report on *Plum bark necrosis and stem pitting-associated virus* infecting grapevine in China.

**Objective:**

The leaf samples of grapevine cultivar 'Cabernet Gernischt' were collected from Shandong province, which the leaves suffered from viral-like symptoms with spotting and crinkle.

**Methods:**

Small RNA-seq combined with reverse transcription PCR (RT-PCR) were performed to detect the potential viruses in these field samples. Phylogenetic tree was constructed using the neighbor joining method in MEGA 5.1

**Conclusions:**

This is the first report of PBNSPaV infecting grapevine in China, contributing to a better understanding of the epidemiology and host range distribution of this pathogen.

## Introduction

Grapevine virus diseases affect the yield and quality of grape (*Vitis vinifera*) [1]. However, with development of grape facility cultivation, especially the seedings were dispatching frequently among different areas, virus diseases have more serious. At present, sixty-six species of viruses were reported to infect grapevine, and some of them can cause serious economic losses worldwide [2–6]. So far, fourteen virus species have been reported in China [4–6]. The diagnostic methods of the virus mainly including biological assay, serum-testing, molecular biology and election microscopy. Meanwhile, one of the primary detection method of small RNA-seq combined with RT-PCR technique was applied, while the primers were designed according to the species-specific virus sequences.

In September 2017, the leaves with symptoms spotting and crinkle were found in Shandong province from the grapevine (*Cabernet Gernischt*), which one of the most common wine grape varieties in China. To detect the potential viruses in these samples, total RNA was extracted by TRIzol reagent and small RNA-seq was performed on the field samples. The FASTQ files of the small RNA HTS have been deposited into GEO and its accession number is PRJNA659879. We acquired 23,725,796 clean reads and 1,868 scaffolds were de novo assembled by velvet (selecting 20-24nt, Kmer = 15). The scaffolds with length more than 50 bp were annotated by BLASTN (identity ≥ 80% and coverage ≥ 80%). Using assembled scaffolds mapped against the viral reference database (ftp://ftp.ncbi.nlm.nih.gov/refseq/release/viral/viral.1.1.genomic.fna.gz) from NCBI and identified 42 scaffolds associated with the following seven viral genomes (identity ≥ 80% and coverage ≥ 80%): *Grapevine virus A* (GVA) (2 scaffolds), *Tomato leaf curl mali virus* (ToLCMLV) (1 scaffolds), *Tomato pseudo-curly top virus* (TPCTV) (1 scaffolds), *Turnip curly top virus* (TCTV) (1 scaffolds), *Prune dwarf virus* (PDV) (11 scaffolds), *Prunus necrotic ringspot virus* (PNRSV) (22 scaffolds), and *Plum bark necrosis* *stem pitting-associated virus* (PBNSPaV) (4 scaffolds).

**Fig. 1:**
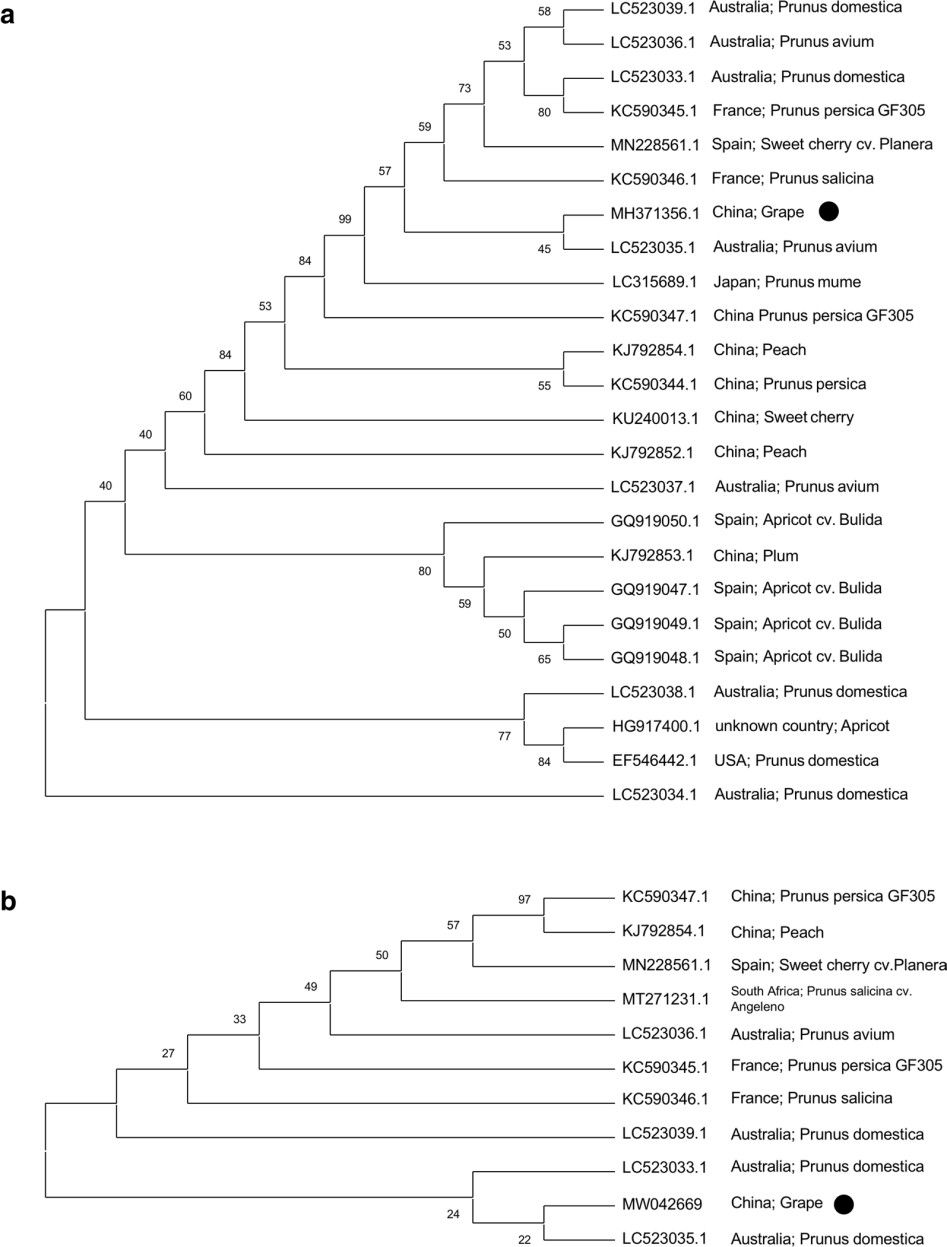
Construction of the phylogenetic trees using the neighbor joining method in MEGA 5.1. **a** Phylogenetic tree was constructed based on the 13,568 bp to 14,139 bp of PBNSPaV; **b** Phylogenetic tree was constructed based on the 9,566 bp to 10,136 bp of PBNSPaV; Bootstrap analysis with 1000 replicates. The new isolate was highlighted with dots

To confirm the presence of the seven viruses in these grapevines, seven pairs of specific primers were utilized to detect the symptomatic samples by RT-PCR and DNA sequencing, which showed that amplified products have the expected sequence size of GVA and PBNSPaV in the samples. And two pairs of primers (PBN-13558-F/PBN-14116-R and PBN-9508-F/PBN-10170-R) (Table 1) were designed to amplify two ~ 550 bp fragments of PBNSPaV. The amplified PCR products were cloned and sequenced using the universal primers M13F and M13R. BLAST analysis showed that the sequences have ~ 98% identities with PBNSPaV (LC523035), which was reported to infect Prunus salicina in Australia [7]. The ~ 550 bp sequences of PBNSPaV from grapevine in this study were submitted to GenBank with the accession number MH371356 and MW042669 respectively. Phylogenetic trees were constructed using MEGA 5.1 software packages and suggested that PBNSPaV (MH371356 and MW042669) have the nearest relationship with the isolate from Australia (LC523035) (Fig. 1). PBNSPaV is a viral species that belongs to the genus *Ampelovirus*, family *Closteroviridae* and has been shown to be worldwide distributed, affecting a broad range of Prunus species [5, 8, 9]. To our knowledge, this is the first report of PBNSPaV infecting grapevine in China. More attention will be paid on the early detection of PBNSPaV, on performance and quality of the grape.

**Table 1:** Primers of RT-PCR for amplifying PBNSPaV

No	Virus	Primer	Sequence
1	PBNSPaV	PBN-9508-F	5-AGTTTTGTTTTCACTGCATGTAG-3
		PBN-10170-R	5-CAACCTGAAACGAGTGGAAC-3
		PBN-13568-F	5-GGATTAGGTGAGGTGTGGTTGAC-3
		PBN-14139-R	5-GTGCATTGCCGATTCCCGGAC-3

## Discussion

As one of the most important grape varieties around the world, 'Cabernet Gernischt' was planted in many wine regions. Shandong is one of the main region of wines, which is the main planting base of the grape. Virus disease is one of the main diseases in grapevine, and may affect the yield and quality of grapevine. PBNSPaV was reported for the first time infecting plum trees (*Prunus salicina Lindl*.) in Dinuba, CA, U.S.A. [10]. Since then PBNSPaV has been shown to be worldwide distributed, affecting a broad range of cultivated and ornamental Prunus species and causes decline, gummosis, flattening of scaffold branches, and stem necrotic pits in some diseased trees [9]. Up to now, there have been no reports of PBNSPaV infecting grape in China. In this study, the results show that PBNSPaV and the associated disease may occur in main cultivated grape species in China. Given the importance and the devastating symptoms of the disease, our findings contributed to a better understanding of the epidemiology and host range distribution of this pathogen.

## Conclusion

In September 2017, the leaf samples of grapevine cultivar 'Cabernet Gernischt' were collected from Shandong province, which the leaves suffered from viral-like symptoms with spotting and crinkle. To detect the potential viruses in these samples, small RNA-seq combined with reverse transcription PCR (RT-PCR) were performed on the field samples, and *Grapevine virus A* (GVA) and *Plum bark necrosis stem pitting-associated virus* (PBNSPaV) were identified. And, this is the first report of PBNSPaV infecting grapevine in China.

## Methods in detail

The total RNA was extracted from infected leaves according to the method of Massart et al. [11] and mixed 3 samples to construct virus sRNA library, and then performed Illumina HiSeq2500 sequencing. The original FASTQ file data should be removed by primers and joint sequences, and after the quality inspection and length screening of the sequencing fragment bases. The sequencing fragment with reliable quality named clean reads. Using cutadapt (version 1.7.1) [12] remove the joint sequence, and filter the sequence length, remove the sequence length less than 15 bp, and the sequence length greater than 41 bp. Fastx_toolkit (version 0.0.13) software, to Q20 quality control sequence, reserve the sequence Q20 reached 80% or more. High quality clean reads were finally obtained by The NGSQCToolkit (Version 2.3.2) [13] and used for subsequent analysis. The transcript sequences were assembled from scratch with clean reads selected 20–24 nt using velvet [14, 15] software. If there is a host reference genome, clean reads is first mismatched with the host, and 20–24 bp sequences that are not matched with the reference genome are selected for assembly. A total of 1,868 scaffolds greater than 50 bp were obtained by using 15 as the Kmer value. All 1,868 scaffolds obtained were annotated with BLASTN software for NT databases on the condition that (identity ≥ 80% and coverage ≥ 80%) only 55 scaffolds annotated with viruses. Virus-related transcripts were obtained by comparison with NT databases. With the refSeq viral genome database (ftp://ftp.ncbi.nlm.nih.gov/refseq/release/viral/viral.1.1.genomic.fna.gz) of the virus through comparing BLASTN homology, if on the level of nucleic acid or protein level high homology (identity ≥ 80% and coverage ≥ 80%) of the transcript is probably from the virus.

## Data Availability

This two sequences of PBNSPaV from grapevine in this study were submitted to GenBank with the accession number MH371356 and MW042669.

## Abbreviations

RT-PCR: Reverse transcription PCR
GVA: Grapevine virus A
PBNSPaV: *Plum bark necrosis stem pitting-associated virus*
NJ: The neighbor-joining method
ToLCMLV: *Tomato leaf curl mali virus*
TPCTV: *Tomato pseudo-curly top virus*
TCTV: *Turnip curly top virus*
PDV: *Prune dwarf virus*
PNRSV: Prunus necrotic ringspot virus
